# Midterm results of left atrial bipolar radiofrequency ablation combined with a mitral valve procedure in persistent atrial fibrillation

**Published:** 2010-06

**Authors:** Hayrettin Tekumit, Kemal Uzun, Ali Riza Cenal, Cenk Tataroglu, Esat Akinci, Adil Polat

**Affiliations:** Avrupa Safak Hastanesi, Istanbul, Turkey; Avrupa Safak Hastanesi, Istanbul, Turkey; Avrupa Safak Hastanesi, Istanbul, Turkey; Avrupa Safak Hastanesi, Istanbul, Turkey; Avrupa Safak Hastanesi, Istanbul, Turkey; JFK Hospital, Istanbul, Turkey

**Keywords:** radiofrequency ablation, atrial fibrillation, mitral valve disease

## Abstract

**Introduction:**

The aim of the study was to assess the midterm results of left atrial bipolar radiofrequency ablation combined with a mitral valve procedure in patients with mitral valve disease and persistent atrial fibrillation.

**Methods:**

Between October 2006 and July 2009, 95 patients with mitral valve disease and persistent atrial fibrillation underwent a mitral valve procedure and left atrial bipolar radiofrequency ablation. The postoperative data of the combined procedure were collected at the time of discharge and at one, three, six and 12 months after the operation.

**Results:**

Hospital mortality rate was 6.3% (six patients). Normal sinus rhythm was achieved in 77.2% of patients during the early postoperative period in hospital, and in 73.3, 72.0 and 75% of patients at three, six and 12 months postoperatively, respectively. Patients were followed up for a mean duration of 14.02 ± 5.71 months (range: 6–19 months). During this midterm follow-up period, nine patients had late recurrence of atrial fibrillation. No risk factor was identified for late recurrence of atrial fibrillation.

**Conclusion:**

Our midterm follow-up results suggest that the addition of left atrial bipolar radiofrequency ablation to mitral valve surgery is an effective and safe procedure to restore sinus rhythm in patients with chronic atrial fibrillation.

## Summary

Atrial fibrillation (AF) is a rapid and irregular activation of the atria so that the normal sinus rhythm disappears. Currently, it is the most frequent form of persistent arrhythmia. Although its incidence ranges from 0.4 to 2% in the general population, this rate is about 10% among individuals over 60 years of age. Atrial fibrillation is particularly common in patients with mitral valve disease (30 to 84%) but is also detected in about 5% of patients with aortic valve and coronary artery disease.^1^ Although most patients with persistent AF have underlying cardiovascular disease, about 31% do not have cardiovascular disease.^2^ Atrial fibrillation may cause heart failure, thromboembolic complications, increased treatment costs and impaired quality of life, and it represents a significant risk for mortality even after the underlying cardiovascular disease is treated.^3^

Medical treatment has been reported to be unsuccessful in approximately 50 and 84% of patients with permanent AF.^4^ Therefore various surgical techniques, including left atrial isolation, catheter ablation of the bundle of His, corridor procedure, pulmonary button isolation and the atrial compartment operation have been adapted to treat atrial fibrillation.^5^ The Cox-Maze III operation, which has become the gold-standard for surgical treatment of atrial fibrillation, with nearly 100% success rates was developed by James Cox and colleagues.^6^ It involves the cutting and sewing of various parts of both atria in order to block the spread of irregular electrical activity by creating lines of isolation in the atrial musculature. Unfortunately, this method needs experience, since it is complex and time-consuming, and also has a high rate of complications.

In recent years, alternative energy sources such as radiofrequency (RF), microwave, laser, bipolar cauterisation and cryoablation have been developed to create isolating lines without cutting the tissue, and thus making ablation easier. Currently, the radiofrequency ablation technique, which was first performed by Sie *et al.* in 1995,^7^,^8^ and cryo-ablation have been the most commonly used methods.

The aim of this study was to assess the midterm results of left atrial bipolar radiofrequency ablation combined with a mitral valve procedure in patients with mitral valve disease and persistent atrial fibrillation.

## Methods

The study included a total of 95 patients (27 male: 27.8%, 68 female: 72.2%; age range: 20–77 years) who underwent a left atrial radiofrequency ablation procedure combined with mitral valve surgery between October 2006 and July 2009. The procedure was performed in all patients with persistent atrial fibrillation (lasting at least six months) plus mitral valve disease, except for AF cases with slow ventricular response. Standard 12-lead electrocardiography, Holter ECG, transthoracic echocardiography, left and right heart catheterisation and coronary angiography were performed before surgery in patients over 40 years old.

Besides symptoms secondary to valve disease, palpitations were present in 70.8% of patients. Sixty-three patients had isolated mitral valve disease. In addition to mitral valve disease, 14 had coronary artery disease, 14 had aortic valve disease, and four had coronary artery disease plus aortic valve disease. Tricuspid valve disease was also present in 31 (33%) of the 95 cases. The aetiology of mitral valve disease was rheumatic fever in 73.8% of the patients. Pre-operative data are summarised in Table 1.

**Table 1.** Baseline Demographical And Clinical Characteristics

*Parameter*	*n = 95*
Gender, M/F (%)	27/68 (27.8/72.2)
Age, mean ± SD	57.38 ± 11.59
Functional capacity, *n* (%)	
NYHA class I–II	11 (11.4)
NYHA class III	62 (65.8)
NYHA class IV	22 (22.8)
Chronic obstructive pulmonary disease, *n* (%)	7 (7.6)
Palpitations, *n* (%)	67 (70.8)
Distribution of structural cardiac pathologies, *n* (%)	
Isolated mitral valve disease	63 (66)
Mitral plus aortic valve disease	14 (15)
Mitral valve plus coronary artery disease	14 (15)
Mitral plus aortic valve disease plus coronary artery disease	4 (4)
Type and aetiology of mitral valve lesions, *n* (%)	
Lesion type	
Mitral stenosis	22 (23)
Mitral insufficiency	28 (30)
Mixed disease	45 (95)
Aetiology	
Rheumatic	70 (74)
Degenerative	17 (18)
Ischaemic	8 (8)
Surgical procedures, *n* (%)	
solated MVR	59 (62)
MVR plus AVR	14 (14)
MVR plus CABG	6 (7)
Isolated mitral valve repair	4 (5)
Mitral valve repair plus CABG	8 (9)
MVR plus AVR plus CABG	4 (5)

NYHA: New York Heart Association; MVR: mitral valve replacement; AVR: aortic valve replacement; CABG: coronary artery bypass grafting.

## Surgical technique

After performing a median sternotomy, aortic and bicaval venous cannulation was performed. Antegrade blood cardioplegia was used for induction, and continuous retrograde blood cardioplegia via the coronary sinus was used for maintenance for myocardial protection. Mitral valve replacement was performed in 83 patients, mitral valve repair in 12, and coronary artery bypass grafting in 18 (Table 1). Mean duration of cross clamping and perfusion times were 84 ± 34 and 113 ± 37 minutes, respectively. To decrease the risk of intracavitary thrombus formation, a continuous heparin infusion was administered until six hours before surgery.

For bipolar left atrial radiofrequency ablation, the Cardioblate® BP2 surgical ablation device (Medtronic model 60831) with serum irrigation was used. Two ablating inserts were mounted on the opposing faces of the jaws of a stainless steel clamp. Each insert was made of two RF electrodes embedded in a polyester covering. The electrodes had a thermocouple mounted on each end. RF current was delivered for 40 to 45 seconds at 35–40 W, with a preset temperature of 90°C. All the ablations were performed on full cardiopulmonary bypass at 32°C.

**Fig. 1.**
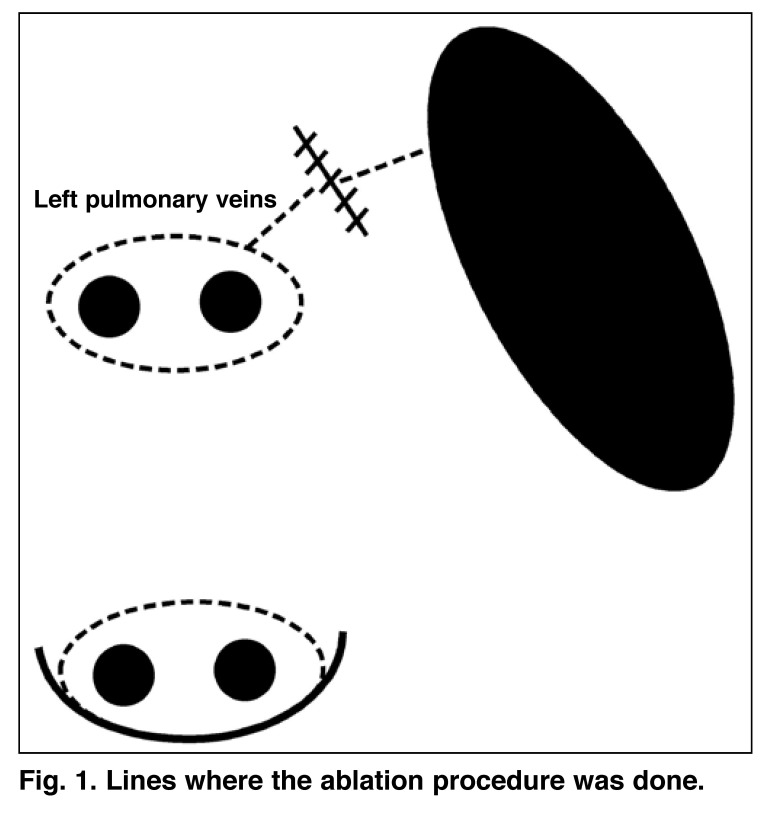
Lines where the ablation procedure was done.

The left atrium was opened following cross clamping. Particular caution was exercised to open a large atriotomy. If mitral valve replacement was to be performed, the mitral valve was removed first and then ablation was done. Firstly, a lesion was created around both right pulmonary veins and the line was joined with a left atriotomy incision, by placing one electrode of the bipolar catheter on the epicardial surface and the other on the endocardial surface of the left atrium. The left pulmonary veins were explored and released, epicardial bipolar ablation was done and both pulmonary veins were isolated as an island. The left atrial appendage (LAA) was resected from outside. Then lesions were created at the lines between (1) the left pulmonary vein lesion and the LAA, and (2) the LAA and the mitral annulus, by placing one electrode of the catheter on the epicardium and the other on the endocardium (Fig. 1). The left atrial appendectomy was sutured from outside following the ablation. Finally, the mitral valvular procedure was performed.

## Follow up

Oral anticoagulation was administered to all patients for three months. Oral anticoagulants were discontinued on the third month postoperatively if mitral and/or aortic valve replacement was not performed, patients were in normal sinus rhythm, or bi-atrial contraction was present.

Anti-arrhythmic prophylaxis was carried out on a routine basis. Amiodarone was used as the first drug of choice: an intravenous bolus of 300 mg, followed by a continuous infusion of 1 200 mg/24 hours was routinely administered until the first postoperative day. In the absence of AV block other than first degree or in the absence of unstable SR, oral administration of 200 mg amiodarone three times a day was given until discharge. A maintenance regimen of 200 mg/day was given for three to six months.

In cases of contraindications to amiodarone, sotalol or propafenone were given. Sotalol plus amiodarone, or electromechanical cardioversion was performed in patients with relapsing AF during hospitalisation. Monitoring with standard 12-lead electrocardiography, Holter ECG and transthoracic echocardiography was done one, three, six and 12 months after the operation.

## Statistical analysis

SPSS (Statistical Package for Social Sciences) for Windows version 10.0 was used for the analysis of data. Besides descriptive statistics (mean ± standard deviation), the Student’s *t*-test and Mann-Whitney U-test were used for the comparison of quantitative data. For the comparison of qualitative data, the Chi-square, Fischer’s Exact Chi-square and Mc Nemar tests were used. Result were evaluated at 95% confidence intervals and at a significance level of *p* < 0.05.

## Results

Hospital mortality was 6.3% (six patients): five due to low cardiac output and one due to pulmonary complications. No morbidity related to the ablation procedure itself was observed. Seven patients had pulmonary complications (7.6%) and nine (10.1%) stayed in the intensive care unit for more than 48 hours. Mean durations of intubation and hospitalisation were 12.27 ± 4.78 hours and 8.7 ± 5.83 days, respectively. No neurological event was observed.

On arrival in the intensive care unit after the operation, 74 patients (77.9%) were in normal sinus rhythm, 15 (15.8 %) were in AF, and six (6.3%) were in AV block. Among the six patients who died in hospital, two of them were in sinus rhythm, two in complete AV block and two in AF. Among the remaining four patients with complete AV block on arrival in the intensive care unit, one developed AF and three resumed normal sinus rhythm.

Sixteen of the patients with normal sinus rhythm on arrival in the intensive care unit relapsed into AF during hospitalisation. Medical treatment with amiodarone and sotalol resumed normal sinus rhythm in five patients, and electromechanical cardioversion resumed normal sinus rhythm in one of these 16 patients. When the 89 surviving patients were discharged, 65 (65/89, 73%) were in normal sinus rhythm and 24 were in AF (24/89, 27%).

**Fig. 2.**
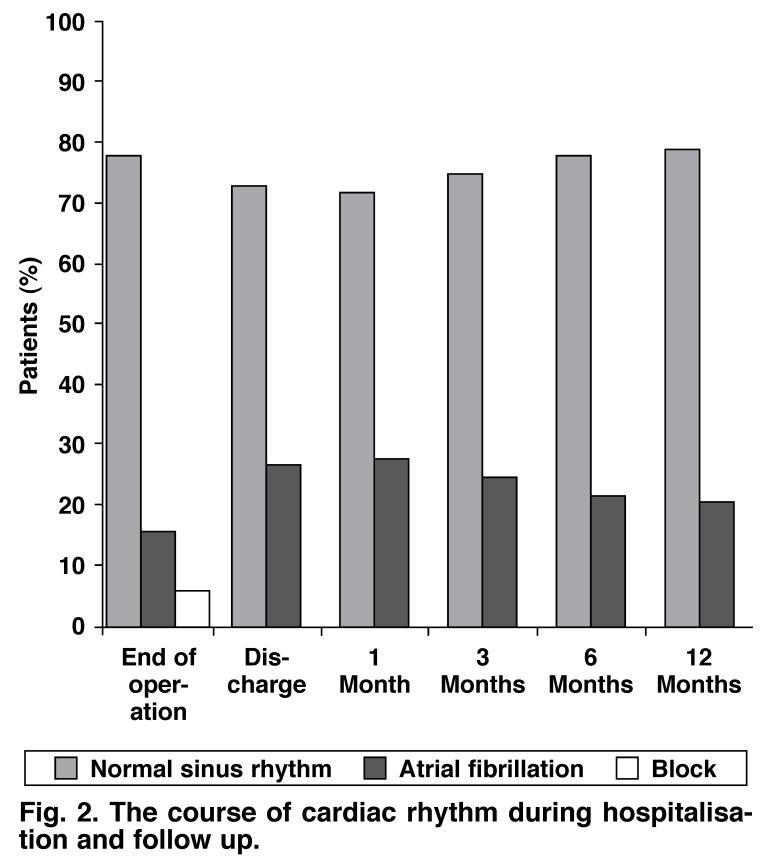
The course of cardiac rhythm during hospitalisation and follow up.

None of the patients required permanent pacemaker implantation during the follow-up period. Of the 89 patients who were discharged, follow up was performed in all at one month, in 81 at three months, in 76 at six months and in 73 at 12 months post surgery (Fig. 2). Unfortunately, the socio-economic and geographical characteristics of our country prevented 100% follow up of the patients.

According to the parameters reviewed at six months postoperatively, patients with atrial fibrillation and sinus rhythm did not differ in terms of left atrial diameter, left ventricular enddiastolic and end-systolic diameter, ejection fraction, pulmonary artery pressure and left ventricular end-diastolic pressure. When the subgroup of patients operated for mitral stenosis was examined, patients with AF and sinus rhythm were similar with regard to mitral valve area and maximum gradients (*p* > 0.05) (Tables 2, 3).

**Table 2.** Distribution Of Af Cases By Demographic Properties

	*Holter-ECG at 6 months*				
	*NSR (n = 59) (mean ± SD)*		*AF (n = 17) (mean ± SD)*		*Test statistics*
Age	60.12 ± 8.23		55.07 ± 12.34		*t*: 1.555; *p*: 0.125
BMI	22.99 ± 6.57		25.58 ± 5.11		*t*: –1.539; *p*: 0.130
Gender	n	%	n	%	
Female	44	70.5	14	82.4	χ^2^: 0.897; *p*: 0.344
Male	15	29.5	3	17.6	

NSR: normal sinus rhythm; AF: atrial fibrillation; *t*: Student’s *t*-test statistics; χ^2^: Chi-square statistics; BMI: body mass index.

**Table 3.** Effects Of Pre-Operative Cardiac Parameters On ECG Rhythm At 6 Months

	*Holter-ECG at 6 months*		
	*AF (n = 17) (mean ± SD)*	*NSR (n = 59) (mean ± SD)*	*Test statistics*
LA	5.11 ± 0.74	4.98 ± 0.63	*z*: –0.422; *p*: 0.673
LVEDD	4.75 ± 1.30	5.06 ± 0.70	*t*: –1,192; *p*: 0.238
LVESD	3.38 ± 0.78	3.25 ± 0.81	*t*: 0.534; *p*: 0.596
EF	55.12 ± 9.79	55.07 ± 9.46	*t*: 0.017; *p*: 0.987
PAP	50.56 ± 14.16	46.70 ± 12.22	*t*: 1.036; *p*: 0.304
LVEDP	9.00 ± 5.24	10.77 ± 11.77	*t*: –0.557; *p*: 0.580
MAXG*	18.75 ± 2.81	17.43 ± 5.59	*t*: 0.633; *p*: 0.532
MVA*	1.55 ± 0.92	1.20 ± 0.81	*z*: –0.773; *p*: 0.440

AF: atrial fibrillation; NSR: normal sinus rhythm; *t*: Student’s *t*-test; *z*: Mann-Whitney U-test; LA: left atrial diameter; LVEDD: left ventricular end-diastolic diameter; LVESD: left ventricular end-systolic diameter; EF: ejection fraction; PAP: pulmonary artery pressure; LVEDP: left ventricular end-diastolic pressure; MAXG: maximum gradient at mitral valve; MVA: mitral valve area. *Patients with mitral stenosis.

Pre-operative functional capacity, type and aetiology of the mitral valve lesion, presence of coronary artery disease, ablation technique, presence of hypertension or the type of mitral intervention (replacement or repair) did not affect the success of the ablation procedures (Table 4).

**Table 4.** Effect Of Pre-Operative Functional Capacity, Type Of Mitral Lesion And Aetiology, Presence Of Coronary Artery Disease, Hypertension, Ablation Technique And Mitral Procedure

		*Holter-ECG at 6 months*				
		*AF (n = 17)*		*NSR (n = 59)*		**
		n	%	n	%	*Test statistics*
NYHA class	II	3	17.6	5	8.5	χ^2^: 0.985; *p*: 0.611
	III	11	64.7	45	76.2	
IV	3	17.6	9	15.3		
Mitral lesion	Stenosis	5	29.4	10	17	χ^2^: 2.126; *p*: 0.547
	Insufficiency	6	35.3	19	32	
	Mixed	6	35.3	30	51	
Mitral aetiology	Rheumatic	12	70.5	49	83	
	Ischaemic	1	5.8	2	3	
	Degenerative	4	23.7	8	14	
Coronary artery disease	Present	6	35.3	18	30	χ^2^: 0.189; *p*: 0.664
	Absent	11	64.7	41	70	
Hypertension	Present	9	52.9	29	49	χ^2^: 0.042; *p*: 0.837
	Absent	8	47.1	30	51	
Mitral procedure	Repair	5	29.5	4	7	Fχ^2^ *p*: 0.080
	Replacement	12	70.5	55	93	

AF: atrial fibrillation; NSR: normal sinus rhythm; χ^2^: Chi-square test.

## Discussion

Most patients with persistent AF have underlying cardiovascular disease and are candidates for open-heart surgery. These patients are prone to develop thromboembolic complications and their cardiac performance is worse than patients with normal sinus rhythm. In more than 80% of patients with mitral valve disease and persistent AF who underwent isolated mitral valve intervention during the cardiac operation, their AF did not resolve.^9^ Surgical treatments for AF, including the Cox-Maze III procedure, a relatively complicated procedure based on a ‘cut and sew’ principle, and radiofrequency ablation, a relatively simple procedure, have resulted in 70 to 99% success rates.

In the series of Cox *et al.*,^10^ the Cox-Maze III procedure was performed in 346 patients, with an operative mortality of 2%. This was the largest sample using the Cox procedure and AF could be treated in 99% of patients, with only 2% requiring longterm postoperative anti-arrhythmia medication. Thirty-eight per cent of their cases had transient postoperative AF, which was attributed to the peri-operative short atrial refractory period, and did not affect long-term results.^11^ Fifteen per cent of patients required permanent pacemakers after the operation. Based on these results, the Cox-Maze III procedure has been accepted as the gold standard for the treatment of AF.

The success rates of Cox-Maze III procedures reported by other centres have been lower than those reported by Cox *et al.* In most series, mitral valve surgery combined with the Cox-Maze III procedure resulted in 75 to 82% success rates for the treatment of AF, and pacemaker implantation was reported in 2 to 24% of cases.^12^-^15^ These investigators attributed their relatively low success rates to the profile of their patients, which included those resistant to medical treatment and having additional cardiac pathologies requiring open-heart surgery. On the other hand, in a study, Cox *et al*. compared patients with and without concomitant valvular surgery and did not find any difference in terms of incidence of peri-operative atrial arrhythmia.^16^

In order to decrease peri-operative bleeding and shorten cross clamping and cardiopulmonary bypass durations, the original incisions of the Cox-Maze III procedure have been replaced by lesions created by energy sources. Rates of 70 to 90% for absence of atrial AF have been reported with radiofrequency ablation.^17^ In the present study, sinus rhythm was resumed in 75, 78 and 79% of cases at the third, sixth and 12th months postoperatively, respectively. Although these rates were lower than those reported by Cox *et al*., they were similar to other results for Cox-Maze III and radiofrequency ablation procedures. None of our patients required a permanent pacemaker. The ease of the procedure, less time needed and rare requirement of permanent pacemaker are the advantages of radiofrequency ablation over Cox-Maze III, whereas cost is its disadvantage.

Various types of lesions have been defined for radiofrequency ablation.^12^,^18^,^19^ All these lesions involve the complete or almost complete isolation of the pulmonary veins, and excision and exclusion of the left atrial appendage. In addition, they include the prevention of transmission by creating lesions between the left pulmonary veins and the left atrial appendage, and also between the left pulmonary veins and the mitral valve annulus. In addition to these lesions, we also created a bipolar lesion on the left atrial isthmus (between the LAA and mitral annulus). Despite the diversity of these lesion types, similar AF treatment rates were found. Success rates were almost as high as those of the Cox-Maze III procedure.

Inclusion of only the left atrium during the ablation procedure is still a debated issue. As atrial fibrillation originates from the left side and atrial flutter from the right side, most authors advocating the Cox-Maze III procedure suggest that an only left-sided procedure would increase the incidence of atrial flutter and probably also the atrial fibrillation rate.^18^ Nakagawa *et al.* particularly emphasised the importance of the isthmus line between the coronary sinus and tricuspid valve, as it is responsible for most atrial flutters.^20^ On the other hand, several theories indicate that an isolated left atrial procedure may be effective. For example, the origin of focally induced atrial fibrillation is frequently at the right atrium, particularly on the pulmonary veins. Therefore electrical isolation of the pulmonary veins has been suggested to prevent the initiation of atrial fibrillation.^21^

Willams *et al*. compared the results of left-sided and bilateral unipolar radiofrequency ablation but did not find a significant difference (79 and 87%, respectively).^18^ Other investigators have reported successful results with isolated left atrial ablation.^22^,^23^ We performed isolated left atrial ablation in all our patients and restored sinus rhythm without atrial flutter in any during the postoperative period. Therefore we tend to favour isolated left atrial ablation for the future. Although right-atrial interventions do not significantly increase the success of the procedure, they increase the duration of ischaemia and cardiopulmonary bypass and necessitate an additional atrial incision, all of which may pose additional risks.

Complications related to radiofrequency ablation have been due to unipolar procedures. Particularly during left atrial ablation, the oesophagus, circumflex coronary artery or left main bronchus may be injured and bleeding due to left atrial perforation may develop. Among these, oesophageal injury is the most fatal.^24^ Another disadvantage of the unipolar catheter is the definitive lack of transmurality. On the other hand, the bipolar system assures a controlled and definitive transmural lesion, thus excluding this disadvantage. However, no significant difference could be found between the two methods with regard to success rates in treating atrial fibrillation.

The peri-operative presence of AF does not indicate failure of the procedure. In the present study, among the 15 patients discharged with AF, 10 returned to normal sinus rhythm at three months. On the other hand, nine patients discharged with sinus rhythm developed AF within the first three months. This may be critical with regard to the long-term success of the procedure in preventing the recurrence of AF. Therefore postoperative prophylaxis for AF is important. We could provide medical cardioversion in five out of seven patients who postoperatively developed AF despite receiving amiodarone, by adding sotalol. Therefore the amiodarone-plus-sotalol combination rather than amiodarone alone may be effective in preventing postoperative recurrences of atrial fibrillation.

Several investigators reported an association between atrial size and success rate, however this finding has not been confirmed by others.^12^,^19^ In the present study, left atrial diameter was greater in patients with persistent AF, although the difference did not reach statistical significance.

The main finding of this study was the importance of the concomitant ablation procedure in patients with mitral valve disease. Although the results were similar to the previous reports discussed above, this study constitutes an important contribution to the literature on using ablation with partial lesions.

## Conclusion

Isolated left atrial radiofrequency ablation effectively treats AF without significantly increasing the duration of cardiopulmonary bypass and without specific complications due to the procedure itself. This procedure demonstrates that AF may be successfully treated by creating a partial lesion, rather than a complete lesion as in the Cox-Maze III procedure.

## References

[1] Martin A, Schwartz MJ, Kronmal RA, Kosinski AS (1988). Prevalence and significance of atrial fibrillation in coronary artery disease (CASS Registry).. Am J Cardiol.

[2] Kannel WB, Abbott RD, Savage DD, McNamara PM (1982). Epidemiologic features of chronic atrial fibrillation: the Framingham study.. N Engl J Med.

[3] Benjamin EJ, Wolf PA, D’Agostino RB, Silbershatz H, Kannel WB, Lewy D (1991). Impact of atrial fibrillation on the risk of death: the Framingham heart study. Final results.. Circulation.

[4] Lundstrom T, Ryden L (1988). Chronic atrial fibrillation. Long-term results of direct current conversion.. Acta Medica Scand.

[5] Graffigna A, Pagani F, Minzioni G, Salerno J, Vigano M (1992). Left atrial isolation associated with mitral valve operations.. Ann Thorac Surg.

[6] Cox JL, Schuessler RB, Lappas DG, Boieneau JP (1996). An 8½-year clinical experience with surgery for atrial fibrillation.. Ann Surg.

[7] Haissaguerre M, Jais P, Shah DC (1996). Right and left atrial radiofrequency catheter therapy of paroxysmal atrial fibrillation.. J Cardiovasc Electrophysiol.

[8] Sie HT, Ramdal Misier R, Beukema WP (1996). Radiofrequency ablation of atrial fibrillation in patients undergoing mitral valve surgery: first experience.. Circulation.

[9] Handa N, Schaff HV, Morris JJ, Anderson BJ, Kopecky SL, Enriquez-Sarano M (1999). Outcome of mitral valve repair and the Cox Maze procedure for mitral regurgitation and associated atrial fibrillation.. J Thorac Cardiovasc Surg.

[10] Cox JL, Ad N, Palazzo T, Fitzpatrick S, Suyderhoud JP, DeGroot KW (2000). Currrent status of the Maze procedure for the treatment of atrial fibrillation.. Semin Thorac Cardiovasc Surg.

[11] Cox JL, Schuessler RB, Boineau JP (1995). The development of the Maze procedure for atrial flutter and atrial fibrillation.. Adv Card Surg.

[12] Chen MC, Chang JP, Guo GB, Chang HW (2001). Atrial size reduction as a predictor of the success of radiofrequency maze procedure for chronic atrial fibrillation in patients undergoing concomitant valvular surgery.. J Cardiovasc Electrophysiol.

[13] Raanani E, Albage A, David TE, Yau TM, Armstrong S (2001). The efficacy of the Cox/maze procedure combined with mitral valve surgery: a matched control study.. Eur J Cardiothorac Surg.

[14] Kamata J, Kawazoe K, Izumoto H, Kitahara H, Shiina Y, Sato Y (1997). Predictors of sinus rhythm restoration after Cox maze procedure concomitant with other cardiac operations.. Ann Thorac Surg.

[15] Izumoto H, Kawazoe K, Kitahara H, Kamata J (1998). Operative results after the Cox/maze procedure combined with a mitral valve operation.. Ann Thorac Surg.

[16] Cox JL, Ad N, Palazzo T, Fitzpatrick S, Suyderhoud JP, DeGroot KW (2000). The Maze III procedure combined with valve surgery.. Seminars Cardiovasc Surg.

[17] Jahangiri M, Weir G, Mandal K, Savelieva I, Camm J (2006). Current strategies in the management of atrial fibrillation.. Ann Thorac Surg.

[18] Williams MR, Stewart JR, Bolling SF, Freeman S, Anderson JT, Argenziano M (2001). Surgical treatment of atrial fibrillation using radiofrequency energy.. Ann Thorac Surg.

[19] Sie HT, Beukema WP, Misier AR, Elvan A, Ennema JJ, Haalebos MM (2001). Radiofrequency modified maze in patients with atrial fibrillation undergoing concomitant cardiac surgery.. J Thorac Cardiovasc Surg.

[20] Nakagawa H, Lazzara R, Khastgir T, Beckman KJ, McClelland JH, Imai S (1996). Role of the tricuspid annulus and the eustachian valve/ridge atrial flutter: relevance to catheter ablation of the septal isthmus and a new technique for rapid identification of ablation success.. Circulation.

[21] Haïssaguerre M, Jaïs P, Shah DC, Takahashi A, Hocini M, Quiniou G (1998). Spontaneous initiation of atrial fibrillation by ectopic beats originating in the pulmonary veins.. N Engl J Med.

[22] Halkos ME, Craver JM, Thourani VH, Kerendi F, Puskas JD, Cooper WA (2005). Introperative radiofrequency ablation for atrial fibrillation during concomitant cardiac surgery.. Ann Thorac Surg.

[23] Kondo N, Takahashi K, Minakawa M, Daitoku K (2003). Left atrial maze procedure: a useful addition to other corrective operations.. Ann Thorac Surg.

[24] Doll N, Borger MA, Fabricius A, Stephan S, Gummert J, Mohr FW (2003). Esophageal perforation during left atrial radiofrequency ablation: Is the risk too high?. J Thorac Cardiovasc Surg.

